# Novel Hybrid Evolutionary Algorithms for Spatial Prediction of Floods

**DOI:** 10.1038/s41598-018-33755-7

**Published:** 2018-10-18

**Authors:** Dieu Tien Bui, Mahdi Panahi, Himan Shahabi, Vijay P. Singh, Ataollah Shirzadi, Kamran Chapi, Khabat Khosravi, Wei Chen, Somayeh Panahi, Shaojun Li, Baharin Bin Ahmad

**Affiliations:** 1 Geographic Information Science Research Group, Ton Duc Thang University, Ho Chi Minh City, Vietnam; 2 Faculty of Environment and Labour Safety, Ton Duc Thang University, Ho Chi Minh City, Vietnam; 3 Geohazard Department Manager, Samaneh Kansar Zamin (SKZ) Company, Tehran, Iran; 4 Department of Geomorphology, Faculty of Natural Resources, University of Kurdistan, Sanandaj, Iran; 5 Department of Biological and Agricultural Engineering & Zachry Department of Civil Engineering, Texas A & M University, College Station, TX 77843-2117 USA; 6 Department of Rangeland and Watershed Management, Faculty of Natural Resources, University of Kurdistan, Sanandaj, Iran; 7 Department of Watershed Management, Faculty of Natural Resources, Sari Agricultural Science and Natural Resources University, Sari, Iran; 8 College of Geology & Environment, Xi’an University of Science and Technology, Xi’an, 710054 Shaanxi China; 9 Young Researchers and Elites Club, North Tehran Branch, Islamic Azad University, Tehran, Iran; 10 State Key Laboratory of Geomechanics and Geotechnical Engineering, Institute of Rock and Soil Mechanics, Chinese Academy of Sciences, Wuhan, Hubei 430071 China; 11 Department of Geoinformation, Faculty of Geoinformation and Real Estate, Universiti Teknologi Malaysia (UTM), Johor Bahru, Malaysia

**Keywords:** Adaptive Neuro-fuzzy Inference System (ANFIS), Firefly Algorithm (FA), Imperialist Competitive Algorithm (ICA), Flood Modelling, Topographic Wetness Index (TWI), Hydrology, Hydrogeology

## Abstract

Adaptive neuro-fuzzy inference system (ANFIS) includes two novel GIS-based ensemble artificial intelligence approaches called imperialistic competitive algorithm (ICA) and firefly algorithm (FA). This combination could result in ANFIS-ICA and ANFIS-FA models, which were applied to flood spatial modelling and its mapping in the Haraz watershed in Northern Province of Mazandaran, Iran. Ten influential factors including slope angle, elevation, stream power index (SPI), curvature, topographic wetness index (TWI), lithology, rainfall, land use, stream density, and the distance to river were selected for flood modelling. The validity of the models was assessed using statistical error-indices (RMSE and MSE), statistical tests (Friedman and Wilcoxon signed-rank tests), and the area under the curve (AUC) of success. The prediction accuracy of the models was compared to some new state-of-the-art sophisticated machine learning techniques that had previously been successfully tested in the study area. The results confirmed the goodness of fit and appropriate prediction accuracy of the two ensemble models. However, the ANFIS-ICA model (AUC = 0.947) had a better performance in comparison to the Bagging-LMT (AUC = 0.940), BLR (AUC = 0.936), LMT (AUC = 0.934), ANFIS-FA (AUC = 0.917), LR (AUC = 0.885) and RF (AUC = 0.806) models. Therefore, the ANFIS-ICA model can be introduced as a promising method for the sustainable management of flood-prone areas.

## Introduction

Flood is considered as one of the most destructive natural disasters worldwide, because of claiming a large number of lives and incurring extensive damage to the property, disrupting social fabric, paralyzing transportation systems, and threatening natural ecosystems^1,2^. Each year flooding affects around 200 million people and causes economic losses of about $95 billion around the world^3^. In Asia, more than half of flood damages are economic and over 90% of all human casualties are the result of flood occurrence^4^. Iran has witnessed many devastating floods recently, especially in the northern cities of Noshahr (2012), Neka (2013), Behshahr (2013), and Sari (2015), all located in the Haraz watershed^5^, Mazandaran, Iran. Arable lands of 28 villages with an area of 80 ha had been destroyed in Chahardangeh district, Sari city; Also, 150 hectares of agricultural lands were degraded in Klijan Restagh in the same city located in the study area^5^.

Although flood prediction models can be used for evaluation of floods that have occurred in the region, prevention of flooding may not be completely possible due to its complexity^6^. Hence, an important solution to reduce future flood damages is to develop models for flood susceptibility mapping based on the determination of flood-prone regions^5,7^. Furthermore, flood susceptibility maps can display the potential of regions for developmental activities by categorizing the sensitivity of areas to flooding^8^.

Recently, some researchers have studied flood susceptibility mapping (FSM) by Remote Sensing (RS) techniques and GIS using different conventional and statistical models, including frequency ratio (FR)^9^, logistic regression (LR)^10^, spatial multi-criteria evaluation (SMCE)^11^, analytical hierarchy processes (AHP)^12^, weight of evidence (WOE)^13^, and evidential belief functions (EBF)^14^. In this regard, Cao *et al*.^15^ compared the FR and statistical index (SI) approaches for flood modelling in the Xiqu Gully (XQG) of Beijing, China^15^. They found that the FR was more powerful in comparison to the SI method for spatial prediction of floods. Tehrany *et al*. (2017) observed that the WOE method outperformed the LR and FR methods. In addition to the above-mentioned models, recently, various machine learning approaches and their ensembles have also been applied to FSM including neuro-fuzzy^16^, artificial neural networks^17,18^, support vector machines^19^, Shannon entropy^20^, decision trees^21^, Naïve Bayes^22^, random forest (RF)^23^, and logistic model tree^3,21^. Chapi *et al*.^24^ introduced a new hybrid artificial intelligence method called Bagging-logistic model tree (LMT) for flood mapping in Haraz watershed, Iran. They also compared the results of the new proposed model with several soft computing benchmark models including LR, Bayesian logistic regression (BLR), and RF. Their results indicated that the new proposed model outperformed and outclassed the other models. Additionally, the results revealed that the prediction accuracy of BLR was greater than that of the LMT, LR and RF models^24^. Khosravi *et al*.^21^ compared some decision tree algorithms including LMT, Reduced Error Pruning Trees (REPT), NBT, and alternating decision tree (ADTree) for flood mapping in Haraz watershed^21^. The results suggested that the ADTree was a more powerful and robust algorithm compared to the NBT, LMT and REPT algorithms. Overall, these results of literature indicated that, one the one hand, the results of performance of all conventional, statistical and machine learning methods are different from a region to another due to the different geo-environmental factors. On the other hand, the results of flood modelling in a given region are also different for some models. This is due to the difference in the structure and framework of the applied algorithms. Therefore, finding the most appropriate model for detecting the flood-prone areas through comparing different methods and also proposing a new model for every region is of great interest to environmental researchers. Among recent approaches on flood, ANFIS model which is a combination of ANN and fuzzy logic, has become increasingly popular^16,25^. However, in some cases, it has not been able to predict the best weights in the modelling process^26^. Therefore, to address this challenge, it is better to use some evolutionary/optimization algorithms enabling us to re-weigh in order to obtain the maximum performance. Although there are some optimization algorithms with different structures and probability distribution functions (PDF) to find the best weights, selecting the best algorithm for the modelling process is a critical issue in the spatial prediction of natural hazard events such as floods. Hence, new hybrid algorithms should be examined to find the best weights and obtain more reasonable results. In this regard, the current study uses new hybrid algorithms including ANFIS-ICA and ANFIS-FA for spatial prediction of floods in Haraz watershed in the northern part of Iran. Although some optimization and machine learning algorithms have been applied to flood modelling around the world, our optimization methods have not been previously explored for flood assessment. For validation and comparison, several quantitative methods such as the Receiver Operating Characteristic (ROC) curve method as well as statistical analysis methods were selected. Computations were performed using Matlab 2016a and ArcGIS 10.2 software.

## Description of the Study Area

**Figure 1:**
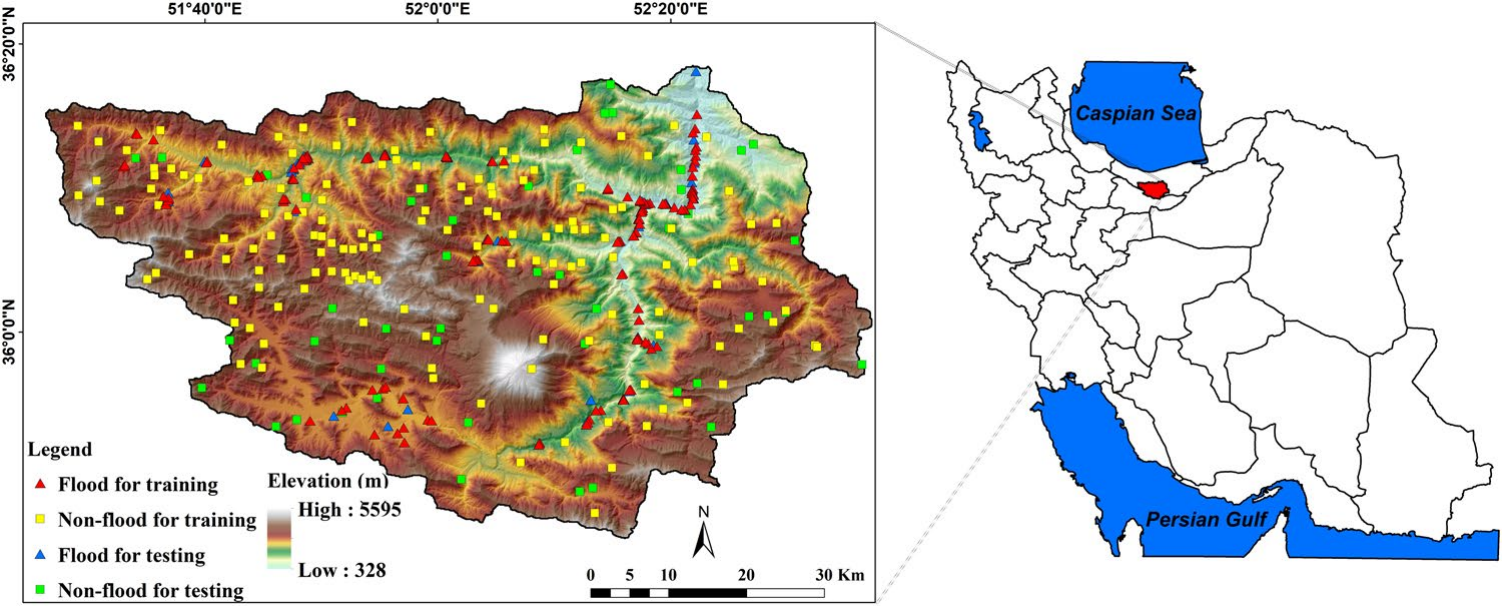
Geographical situation of Haraz watershed and locations of flood and non-flood occurrences in the study area.

Located between longitudes of 51° 43′ to 52° 36′E, and latitude of 35° 45′ to 36° 22′N, Haraz watershed is situated in the south of Amol City in Mazandaran Province, Northern Iran. This watershed drains an area of 4,014 km^2^. Elevation of the watershed ranges from 328 m to about 5595 m at the highest point and its ground slope varies from flat to 66° (Fig. 1). The average annual rainfall of Mazandaran province is 770 mm, and for the Haraz watershed case study, it is 430 mm. The maximum rainfall occurs in January, February, March, and October with an average monthly rainfall of 160 mm (Iran’s Meteorological Organization) during these four months. The climate in the study area is a combination of the mild humid climate of the Caspian shoreline area and the moderate cold climate of mountainous regions. The area is almost entirely encompassed by rocks from Mesozoic (56.4%), Cenozoic (38.9%), and Paleozoic (4.7%) eras. The rangeland covers around 92% of the study area. Other land use patterns present in the area include forest, irrigation land, bare land, garden land, and residential area.

The main residential centres located in Haraz watershed include Abasak, Baladeh, Gaznak, Kandovan, Noor, Polur, Rineh, Tashal, and Tiran^5^. The Haraz watershed was considered for the present study due to the many devastating floods that have occurred annually, claiming lives and incurring damage to the property in recent years. The major reason for flooding in these areas is high rainfall intensity within a short period of time, major land use changes specifically from rangelands to farmlands, deforestation, recent extensive changes from orchards to residential areas, and the lack of control measures required to curb flooding. In the study area, floods lead to fatalities and damages to infrastructure, and disruption of traffic, trade and public services.

## Results and Discussion

### Spatial relationship between floods and influential factors using SWARA model

The first step in this study was determining the sub-factor weights based on the SWARA algorithm, as presented in Table 1. Table republished from^27^. In this study, 10 factors showing a significant impact on flood occurrence were selected based on literature review, the characteristics of watershed, and data availability^5^. One of the most significant factors in flood modelling is the slope angle. The slope map was developed and then classified into seven classes by the natural break classification method. The SWARA value was the highest for the class 0–0.5 (0.4). Hence, the results revealed that the higher the slope angle, the less the probability of flooding will be. Another important factor was elevation which was classified into nine classes. The results showed that the first class, 328–350 m, with a weight of 0.63, had the highest impact on flooding such that the higher the elevation, the lower the flooding probability will be. This was confirmed by Khosravi *et al*.^5^ and Tehrany *et al*.^19^ who explained that slope angle, elevation, and distance to river had higher impacts on flooding such that once the slope angle, elevation, and distance to river increased, the probability of flooding diminished. Meanwhile, curvature is representative of topography of the ground surface. This factor was prepared and classified into three classes including concave, convex, and flat. For curvature, the highest SWARA value was found for concave followed by flat and convex.

**Table 1:** Spatial relationship between flood and conditioning factors of SWARA model. Table republished from Reference^27^.

Factors	Classes	Comparative importance of average value Kj	Coefficient Kj = Sj + 1	wj = (X (j − 1))/kj	weight wj/sigma wj
Slope	0–0.5	—	1.00	1.00	0.40
	0.5–2	0.80	1.80	0.56	0.22
	2–5	0.20	1.20	0.46	0.18
	5–8	0.60	1.60	0.29	0.11
	8–13	1.15	2.15	0.13	0.05
	13–20	1.50	2.50	0.05	0.02
	>30	2.70	3.70	0.01	0.01
	20–30	0.55	1.55	0.01	0.00
Elevation	328–350	—	1.00	1.00	0.63
	400–450	3.70	4.70	0.21	0.13
	350–400	0.35	1.35	0.16	0.10
	450–500	0.55	1.55	0.10	0.06
	500–1000	0.65	1.65	0.06	0.04
	1000–2000	3.95	4.95	0.01	0.01
	2000–3000	0.00	1.00	0.01	0.01
	3000–4000	0.00	1.00	0.01	0.01
	4000>	0.00	1.00	0.01	0.01
Curvature	Concave	—	1.00	1.00	0.46
	Flat	0.05	1.05	0.95	0.43
	Convex	3.00	4.00	0.24	0.11
SPI	2000–3000	—	1.00	1.00	0.32
	800–2000	0.10	1.10	0.91	0.29
	400–800	0.30	1.30	0.70	0.22
	80–400	0.70	1.70	0.41	0.13
	0–80	3.70	4.70	0.09	0.03
	>3000	3.95	4.95	0.02	0.01
TWI	6.96–11.5	—	1.00	1.00	0.08
	5.72–6.96	0.10	1.10	0.91	0.07
	5.03–5.72	0.65	1.65	0.55	0.04
	4.47–5.03	2.70	3.70	0.15	0.01
	3.94–4.47	3.50	4.50	0.03	0.00
	1.9–3.94	0.05	1.05	0.03	0.00
River density	2.67–3.66	—	1.00	1.00	0.00
	3.66–7.3	0.00	1.00	1.00	0.01
	1.92–2.67	0.85	1.85	0.54	0.06
	1.17–1.92	2.50	3.50	0.15	0.20
	0.401–1.17	3.95	4.95	0.03	0.37
	0–0.401	3.95	4.95	0.01	0.37
Distance to river	0–50	—	1.00	1.00	0.59
	50–100	1.75	2.75	0.36	0.22
	100–150	0.85	1.85	0.20	0.12
	150–200	1.20	2.20	0.09	0.05
	200–400	2.70	3.70	0.02	0.01
	400–700	2.70	3.70	0.01	0.00
	700–1000	3.00	4.00	0.00	0.00
	>1000	0.00	1.00	0.00	0.00
Lithology	Teryas	—	1.00	1.00	0.31
	Quaternary	0.50	1.50	0.67	0.21
	Permain	0.00	1.00	0.67	0.21
	Cretaceous	0.40	1.40	0.48	0.15
	Jurassic	1.10	2.10	0.23	0.07
	Teratiary	0.10	1.10	0.21	0.06
Land use	Water bodies	—	1.00	1.00	0.75
	Residential area	3.90	4.90	0.20	0.15
	Garden	1.55	2.55	0.08	0.06
	Forest land	2.00	3.00	0.03	0.02
	Grassland	0.70	1.70	0.02	0.01
	Farming Land	3.95	4.95	0.00	0.00
	Barren land	0.00	1.00	0.00	0.00
Rainfall	188–333	—	1.00	1.00	0.40
	379–409	1.20	2.20	0.45	0.18
	409–448	0.35	1.35	0.34	0.13
	333–379	0.10	1.10	0.31	0.12
	448–535	0.05	1.05	0.29	0.12
	535–741	1.15	2.15	0.14	0.05

The results of SPI revealed that the class of 2000–3000 (0.32) showed a higher impact on flood occurrence than other classes such that higher SPIs were associated with higher flood probability. In the case of topographic wetness index (TWI), the SWARA value showed a decreasing trend when the TWI value increased. The highest SWARA value belonged to the class of 6.96–11.5 (0.08). TWI indicates the wetness of land; wetness was higher in areas with lower slope than mountainous areas. This suggests that larger TWI values are associated with higher flood probability. River density results also showed that the river density values higher than 3.66 had the highest correlation with flood occurrence (0.37). The results of the SWARA model revealed that as the river density grew, the probability of flood occurrence also increased. For distance-to-river factor, the results demonstrated that the first class of 0–50 m (0.59) had a relatively higher susceptibility to flood occurrence. The farther the distance to the river, the less probable the flood occurrence was. For lithology, Teryas formations had the highest SWARA value (0.31) suggesting the highest probability of flood occurrence.

The land use map was constructed for seven types including water bodies, residential area, garden, forest land, grassland, farming land, and barren land. The water bodies demonstrated a higher impact on flood occurrence (0.75), followed by residential area (0.15), garden (0.06), forest land (0.02), grassland (0.01), farming land (0), and barren land (0). The highest SWARA value (0.4) was assigned to the lowest amount of rainfall (188–333), where the higher the rainfall level, the less the flood occurrence probability was. The reason why rainfall increase was still insignificant for flooding was that rainfall increased in areas where elevation increased and these areas were not prone to flooding^5^.

### Sensitivity analysis of conditioning factors

The results of sensitivity analysis are reported in Table 2. Based on the testing error values, the results indicated that in the ANFIS-ICA hybrid model, when the slope angle, elevation, curvature, TWI, SPI, rainfall, distance to river, river density, lithology, and land use were removed, the RMSE values were 0.196, 0.141, 0.172, 0.145, 0.133, 0.143, 0.134, 0.172, 0.145, and 0.134, respectively. It was observed that when all factors were considered in the modelling process, the error value was 0.130. It was also seen that all conditioning factors had a positive role in the flood modelling process. On the other hand, the results of sensitivity analysis for ANFIS-FA hybrid model concluded that with removing the slope angle, elevation, curvature, TWI, SPI, rainfall, distance to river, river density, lithology, and land use factors, the RMSE values were computed as 0.269, 0.196, 0.257, 0.193, 0.173, 0.198, 0.171, 0.260, 0.201, and 0.171, respectively. In this model, when all factors were introduced into the model, the error value was 0.170. Overall, the results suggested that all factors were also significant for flood modelling in ANFIS-FA hybrid model.

**Table 2:** Sensitivity analysis of flood modeling using ANFIS-ICA and ANFIS-FA models.

		Slope angle	Elevation	Curvature	TWI	SPI	Rainfall	Dis-river	River density	Lithology	Land use	All
ANFIS-ICA	Train	0.067	0.065	0.076	0.074	0.089	0.053	0.066	0.076	0.044	0.048	0.069
	Test	0.196	0.141	0.172	0.145	0.133	0.143	0.134	0.172	0.145	0.134	0.130
ANFIS-FA	Train	0.084	0.080	0.094	0.075	0.078	0.063	0.080	0.081	0.052	0.059	0.078
	Test	0.269	0.196	0.257	0.193	0.173	0.198	0.171	0.260	0.201	0.171	0.170

### Application of ANFIS ensemble models

Using the MATLAB programming language, two hybrid models called ANFIS with complementary models of FA and ICA were developed, leading to ANFIS-ICA and ANFIS-FA, which were initially trained. The accuracy of training was then calculated using test data. To that end, the dataset related to flood and non-flood was segmented into 70% for training and 30% for testing. Then, the two methods started extracting and learning the relationship between the SWARA values for conditioning factors and flood (1) as well as non-flood (0) values.

### Model results and validation

**Figure 2:**
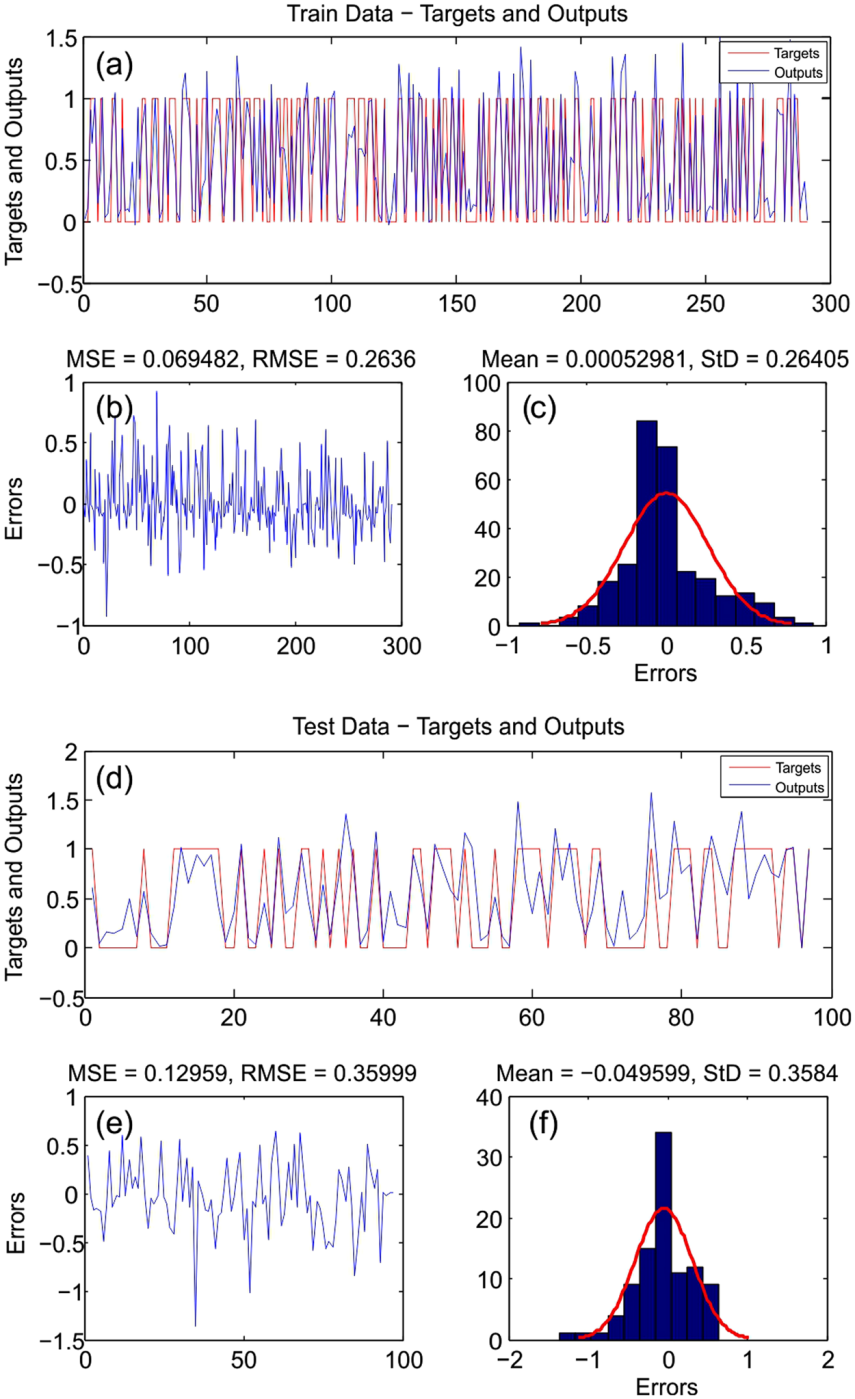
ANFIS-ICA model: (**a**) target and output ANFIS-ICA value of the training data samples; (**b**) MSE and RMSE value of the training data samples; (**c**) frequency errors of the training data samples; (**d**) target and output ANFIS-ICA value of the testing data samples; (**e**) MSE and RMSE value of the testing data samples; and (**f**) frequency errors of the testing data samples.

**Figure 3:**
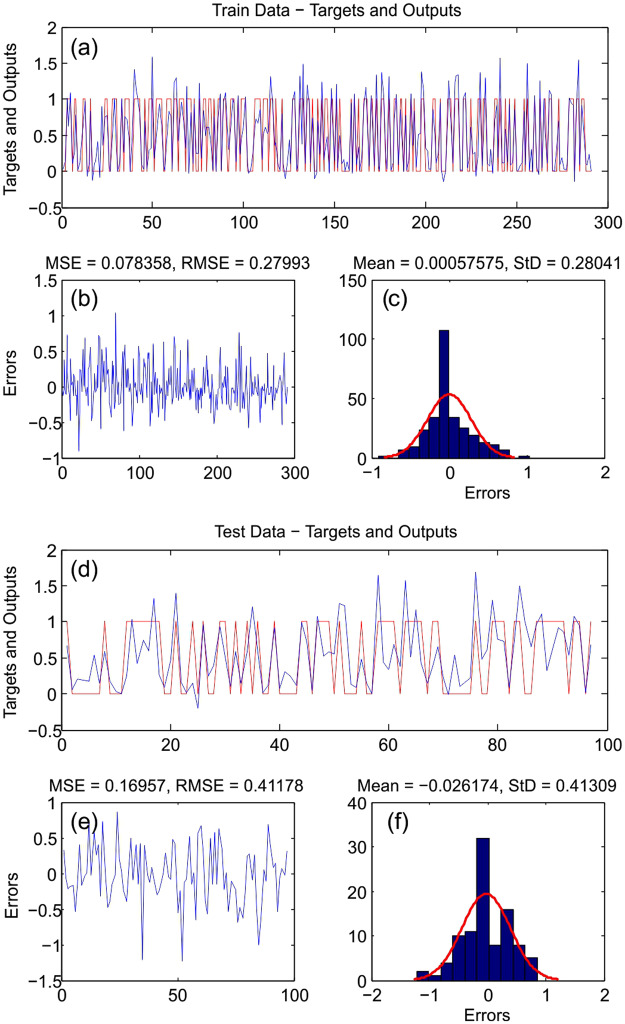
ANFIS-FA model: (**a**) target and output ANFIS-FA value of the training data samples; (**b**) MSE and RMSE value of the training data samples; (**c**) frequency errors of training the data samples; (**d**) target and output ANFIS-FA value of the testing data samples; (**e**) MSE and RMSE value of the testing data samples; and (**f**) frequency errors of the testing data sample.

The results of the model prediction capacity were evaluated using Mean Squared Error (MSE) and Root Mean Squared Error (RMSE) for both modelling and validation^26^. Figures 2 and 3 demonstrate the comparison between the training dataset as a target and estimated flood pixels as the output for modelling by ANFIS-ICA and ANFIS-FA. The MSE and RMSE values for ANFIS-ICA and ANFIS-FA models were 0.069 and 0.26 in the training, and 0.078 and 0.27 in the testing, respectively. Accordingly, ANFIS-ICA had a better performance compared to ANFIS-FA in the training due to lower MSE and RMSE. Note that the optimized model was the model that predicted the results of testing data with higher accuracy. The values of MSE and RMSE for ANFIS-ICA and ANFIS-FA were obtained as 0.41 and 0.35, and 0.169 and 0.129, respectively. Since MSE and RMSE of ANFIS-ICA were lower than of those of ANFIS-FA, it can be concluded that ANFIS-ICA was a more optimal model than ANFIS-FA, since ANFIS-ICA had higher accuracy in both the training and testing.

### Preparation of flood susceptibility mapping using ANFIS ensemble models

In this study, the ensembles of ANFIS based on the ICA and FA algorithms were developed by the training dataset. The developed models were then applied for calculating the flood susceptibility index (FSI) which was assigned to all pixels of the study area to create flood susceptibility maps (FSM). In the first step, each pixel of the study area was assigned to a unique flood susceptibility index; then, these indices were exported in Arc GIS 10.2 format and used to construct the final flood susceptibility mapping.

**Figure 4:**
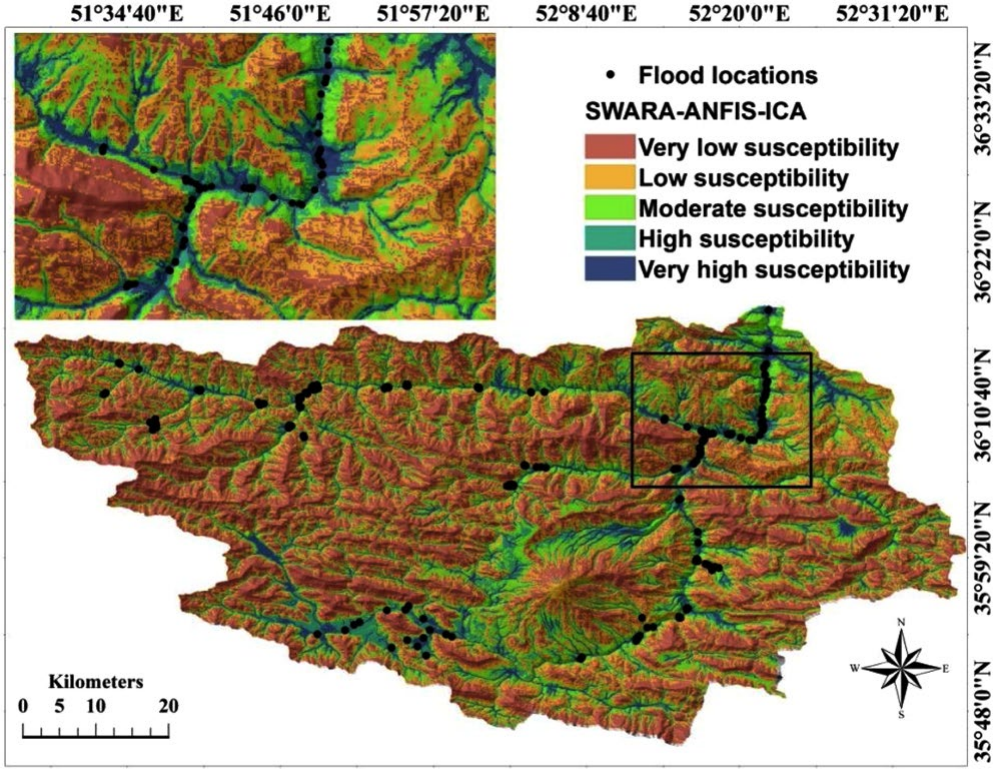
Flood susceptibility mapping prepared via ANFIS-ICA model.

**Figure 5:**
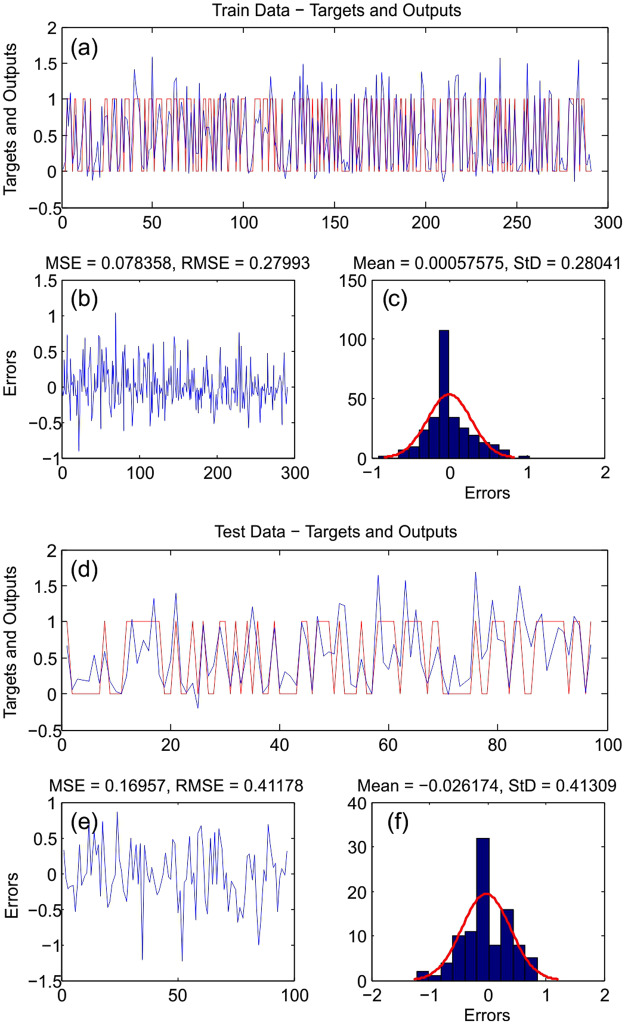
Flood susceptibility mapping prepared by ANFIS-FA model.

There are different techniques for map classification in the Arc GIS 10.2 software including natural break, equal interval, geometrical interval, quantile, standard deviation, and even manual technique. They should be tested to select the most appropriate one to classify any map in the study area. Generally, selection of the classification methods is based on the distribution of flood susceptibility indexes^28^. For example, if the distribution of FSI values is close to normal, to prepare an equal interval, FSM or standard deviation classification methods should be used. However, when FSI has positive or negative skewness, the best classification methods are quantile or natural break^29^. In the current study, the data distribution histogram revealed that quantile classifier was capable of generating better results compared to other classifiers. Thus, the approach of quantile data classification was selected, and FSI was classified in four classes of susceptibility: low, moderate, high, and very high^19^. Finally, based on the hybrid model, the two maps of flood susceptibility were constructed, as depicted in Figs 4 and 5.

### Validation and comparison of new hybrid flood susceptibility models

#### Validation of the proposed ensemble models

**Figure 6:**
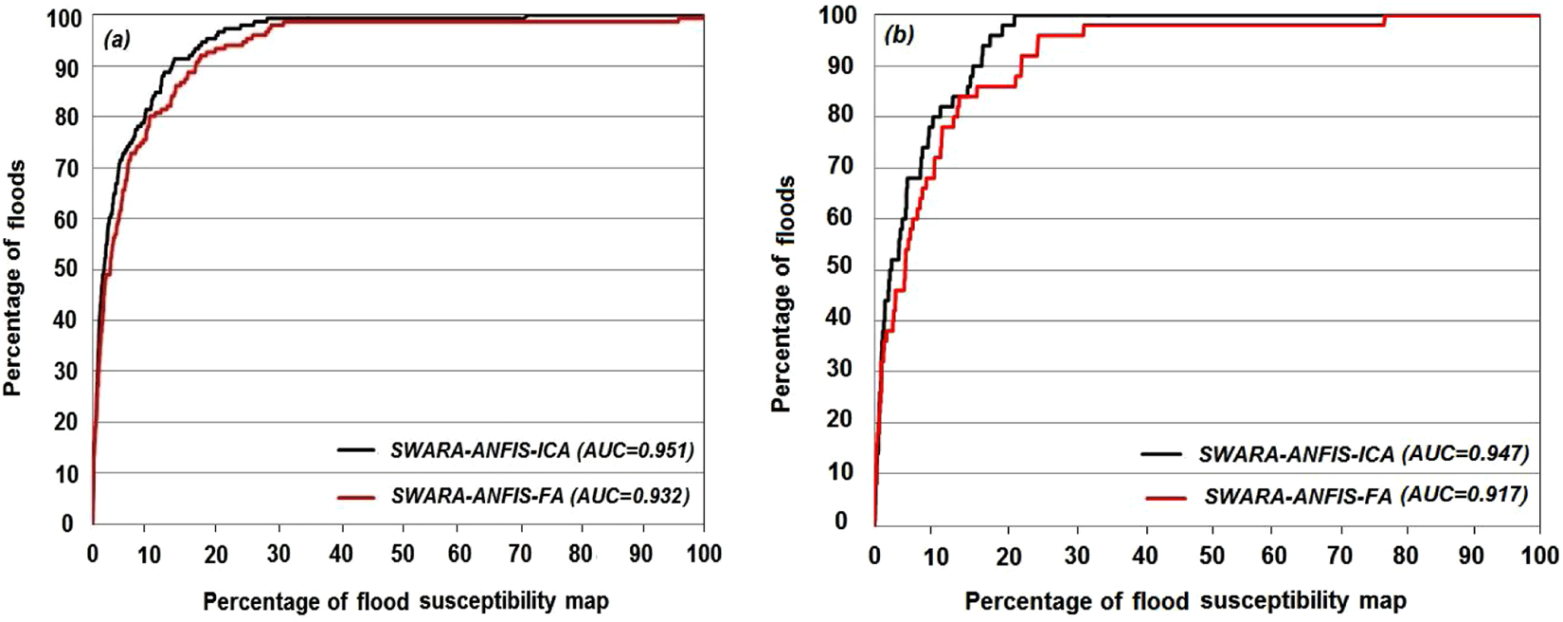
Area under the curves of success rate (**a**) and prediction rate (**b**) of new flash flood hybrid models.

The reliability and predictive power of these two new hybrid models for flood modelling were assessed using success and prediction rate curves. The outcomes of success and prediction rate were prepared for 70% (training data) and 30% (testing data that were not used in training) of data. Since the success rate curve was drawn using the training data, It was impossible to evaluate the predictive power of the models^30^. On the other hand, the AUC for prediction rate curve showed how well the model predicted floods^31^. The area under ROC curve was considered for evaluating the overall performance of the models. According to that, Larger AUC represented better performance of the model. The ROC method has been one of the most popular techniques to evaluate the efficiency of models, as this method quantitatively calculates the efficiency of models^5^. The results of success-rate showed that the AUC values for the two models of ANFIS-ICA and ANFIS-FA were 0.95 and 0.93, respectively (Fig. 6a). The prediction rate which was not used in the modelling was applied to the assessment of the model capacity in predicting flood-prone areas. The values of area under the prediction-rate curve for ANFIS-ICA and ANFIS-FA were 0.94 and 0.91, respectively (Fig. 6b). Then, the highest predictive power for flood-prone areas in the Haraz watershed was provided by the new hybrid ANFIS-ICA model, which had also a lower standard error of 0.35 compared to the ANFIS-FA (0.41) model. The results were compatible with those of the RMSE and MSE values in both the training and testing phases. These two new hybrid models had also a reasonable ROC; yet, the ANFIS-ICA model represented the best performance in predicting the flood susceptibility, followed by the ANFIS-FA model.

In addition to the success and prediction rate curves, Friedman and Wilcoxon signed-rank tests were also utilized for assessing the validity of the two new hybrid models (the performance of the models). They determined whether there were significant statistical differences between performances of the two hybrid models. The results of Freidman test reported in Table 3 indicated that the average ranking values for the ANFIS-ICA and ANFIS-FA hybrid models were 1.29 and 1.71, respectively. Although chi-square value was 35.945, due to having a significant level of less than 5%, the Friedman test was not applicable to assess the validity of models.

**Table 3:** Average ranking of the two flash flood susceptibility hybrid models using Friedman’s test.

No	Flash flood models	Mean ranks	χ^2^	Sig.
1	ANFIS-ICA	1.29	35.945	0.000
2	ANFIS-FA	1.71		

To overcome this challenge, the Wilcoxon signed-rank test was performed for determining the pairwise differences between the two flood hybrid models at the 5% significance level (Table 4). No significant difference was detected between the two flood hybrid models at the significance level of 5% when the null hypothesis was rejected. On the other hand, in this case their results were not the same. The results of Wilcoxon signed-rank test were specified based on the p-value and z-value. If the p-value was less than 5% (0.05), and z-value was larger than −1.96 and +1.96, the performances of the two models were significantly different^32^. The results of Wilcoxon signed-rank test, shown in Table 3, imply that there was a statistical difference between the two flood susceptibility models.

**Table 4:** Performance of the new flash flood hybrid models by Wilcoxon signed-rank test (two-tailed).

No	Pair wise comparison	Number of positive differences	Number of negative differences	z-value	p-value	Significance
1	S-A-ICA vs. S-A-FA	143	58	−3.982	0.000	Yes

(The standard p value is 0.05).

#### Comparison with some state-of-the-art sophisticated machine learning techniques

**Figure 7:**
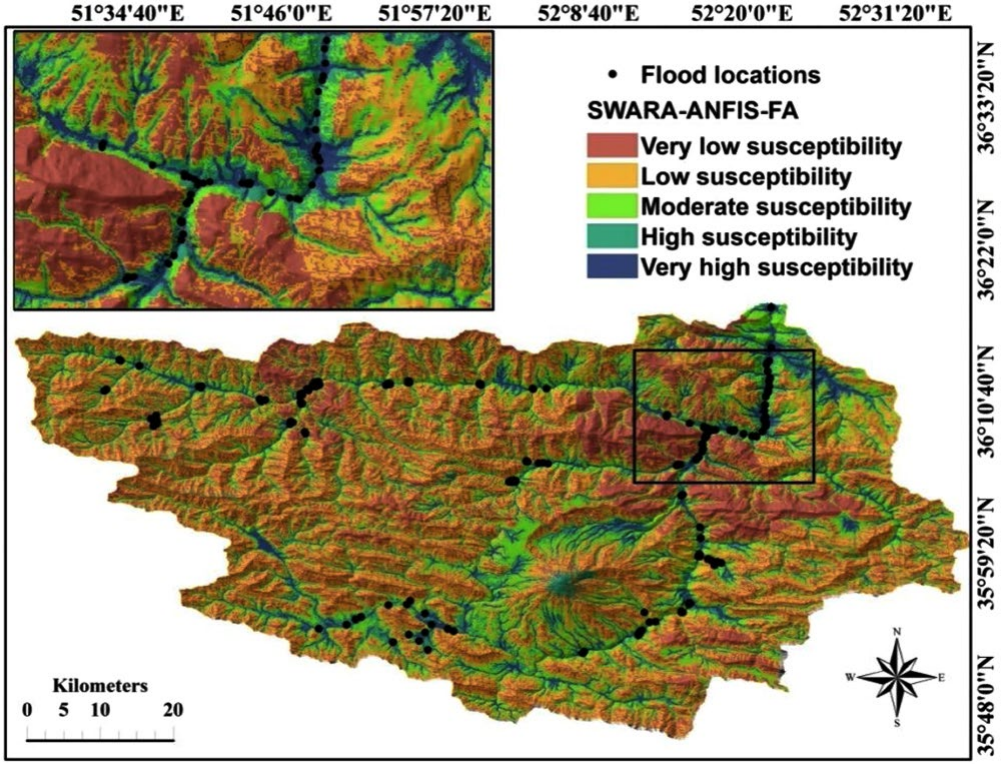
Area under curve of the prediction rate using validation dataset.

Chapi *et al*.^24^ introduced a novel ensemble data mining model called Bagging-logistic model tree (LMT) for mapping of flood susceptibility in this study area. Then, the results of this new model were compared with those of some state-of-the-art sophisticated machine learning models including LMT, Bayesian logistic regression (BLR), logistic regression (LR) and random forest (RF). Comparison in Fig. 7 reveals that the new proposed model outperformed all these models. For the validation dataset, the highest AUC was obtained for the new ensemble model (AUC_Bagging-LMT_ = 0.940), followed by BLR (AUC = 0.936), LMT (AUC = 0.934), LR (AUC = 0.885) and RF (AUC = 0.806). The evolutionary algorithm, ANFIS-ICA (AUC = 0.947) outperformed all these models, but ANFIS-FA was unable to show a greater performance than the Bagging- LMT, BLR and LMT models. However, AUC_ANFIS-FA_ was better than the LR and RF models. Overall, the new proposed model, ANFIS-ICA, was a powerful ensemble model which improved the prediction accuracy among all models in this study area.

## Conclusions

The dynamic nature of floods will always necessitate new approaches and models for its management. This is the main reason that no one has been able to introduce the best model. Unpredicted spatial-temporal changes of this phenomenon have forced scientists to continuously seek for better approaches to generate better results and outcomes; sometimes new individual models and sometimes combinations of the individual models known as hybrid models. In the current study, two new hybrid models, ANFIS-ICA and ANFIS-FA, were applied to enhance the predictive power of flood spatial modelling. A total of 10 conditioning factors were selected for the spatial modelling in the Haraz watershed, Northern Iran. The factors were divided into several classes using the SWARA model and their maps were generated for modelling purposes. The new hybrid models were then used for modelling spatial floods in the study area. The models’ outputs were compared to the results of several soft computing approaches that had been successfully used before in the study area whose accuracy had been approved. The ANFIS-ICA model was the most successful among all the models and its results provided the most appropriate congruence with reality. Although the results of ANFIS-FA were reasonable, the model was not ranked as the best. Accordingly, this study introduced a new model, ANFIS-ICA, for spatial prediction of floods in the study area and other similar watersheds. This model can be evaluated as a tool for more appropriate mitigation and management of floods in Northern Iran.

## Materials and Methods

### Data collection and preparation

Flood inventory maps, which are generated in accordance with historical flood data, are the cornerstones of spatial prediction of floods. The quality of historical data is closely related to the accuracy of the prediction model^33^. Combined with field survey, flood records in 2004, 2008 and 2012 were employed to develop a flood inventory map for the study area. Additionally, DEM (derived from the Advanced Spaceborne Thermal Emission and Reflection Radiometer (ASTER) Global DEM (https://gdex.cr.usgs.gov/gdex/) with a resolution of 30 × 30 m), and geological maps in the Haraz watershed were also used in data collection and preparation. Eventually, 201 flood points and non-flood points were selected utilizing satellite images. In order to build spatial prediction models and to test the validity of model results, these data points were randomly divided into a training (70%; 141 locations) and a testing (30%; 60 locations) set^24^. In the present study, 10 flood conditioning factors were adopted: curvature, distance to river, elevation, land use, lithology, rainfall, river density, SPI, slope, and TWI. These factors were extracted using DEM and geological maps by ArcGIS software.

### Sensitivity analysis

The relative importance of different conditioning factors affecting the modelling process and outputs should be assessed using sensitivity analysis approaches^34,35^. Ilia and Tsangaratos (2016) reported that the sensitivity analysis is performed using three methods including: (i) Changing criteria values, (ii) Changing relative importance of criteria, and (iii) Changing criteria weights^36^. The current study adopted the second method, changing relative importance of criteria, such that each conditioning factor was firstly removed and then modelling was performed with other factors. Accordingly, the obtained results were compared with the condition taking all conditioning factors for the modelling into account by error estimation such as RMSE measure.

### Theoretical background of the methods used

#### Stepwise Assessment Ratio Analysis (SWARA)

The Step-wise Assessment Ratio Analysis (SWARA), which was first proposed by Keršuliene in 2010, is one of the Multi-Criteria Decision Making (MCDM) methods. Experts play an important part in calculating the weights in this method, and hence the SWARA method is called an expert-oriented method^37^. The expert assigns the highest rank to the most valuable and the lowest rank to the least valuable criteria. Subsequently, the average value of these ranks determines the overall ranks. The SWARA method follows these steps^37^:

***Step 1:*** Criteria are developed and determined. The expert must develop decision-making models from the determined factors. Moreover, the criteria are arranged in accordance with their priority and the importance assigned by the expert’s viewpoint and then the influential criteria are sorted in a descending order.

***Step 2:*** criteria’s weighting is calculated. Then, the weights are assigned to all criteria based on expert’s knowledge, information gained for the case study, and the previous experience as follows:

The respondent expresses the comparative importance of the criterion j compared to the previous (j − 1) criterion starting from the second criterion, with the trend repeating the same way for each particular criterion. The trend, according to Keršuliene, determines the relative importance of the average value, S_j_ in this manner^37^:

1 $${S}_{j}=\frac{{\sum }_{i}^{n}\,{A}_{i}}{n}$$

where, n represents the number of experts; A_i_ stands for the ranks suggested by the experts for each factor; and j shows the number of factors involved. The coefficient K_j_ is calculated as:

2 $${K}_{j}=\{\begin{array}{ll}1 & j=1\\ {S}_{j}+1 & \,j > 1\end{array}$$

The recalculation of Q_j_ is done by:

3 $${Q}_{j}=\frac{{X}_{j-1}}{{K}_{j}}\,$$

The relative weights of evaluation criteria can be expressed as:

4 $${W}_{j}=\frac{{Q}_{j}}{{\sum }_{j=1}^{m}{Q}_{j}}\,$$

where, W_j_ denotes the relative weight of the j-th criterion, and m represents the total number of criteria.

#### Adaptive neuro-fuzzy inference systems (ANFIS)

Adaptive Neuro Fuzzy Inference System (ANFIS) is a data-driven model belonging to the family of neuro-fuzzy methods. The ANFIS is a hybrid algorithm integrating neural network with fuzzy logic which generates input-output data pairs^38^. This algorithm, first proposed by Jang in^39^, is used in many fields such as data processing of landslides^40^, flooding^26^, and groundwater^41^. The fuzzy inference system has two inputs x_1_ and x_2_, one output z, the fuzzy set A_1_, A_2_, B_1_, B_2_, and p_i_, q_i_, and r_i_ which are the consequence of output function parameters in a set of fuzzy IF-THEN rules based on the first-order Takagi-Sugeno fuzzy model^42^ as follows:

5 $${\rm{Rule}}\,1:{\rm{if}}\,{x}_{1}\,{\rm{is}}\,{A}_{1}\,{\rm{and}}\,{y}_{1}\,{\rm{is}}\,{B}_{1},\,{\rm{then}}\,{f}_{1}={p}_{1}{x}_{1}+{q}_{1}{y}_{1}+{r}_{1}$$

6 $${\rm{Rule}}\,1:{\rm{if}}\,{x}_{2}\,{\rm{is}}\,{A}_{2}\,{\rm{and}}\,{y}_{2}\,{\rm{is}}\,{B}_{2},\,{\rm{then}}\,{f}_{2}={p}_{2}{x}_{2}+{q}_{2}{y}_{2}+{r}_{2}$$

The ANFIS is a feedforward neural network with a multi-layer structure^43^. The most important point is the parameters of ANFIS which must be optimized by other methods. In this study, the imperialist competitive algorithm (ICA) and the Firefly algorithm (FA) were used for optimization.

#### Firefly algorithm (FA)

As an evolutionary algorithm, Firefly algorithm was first introduced by Yang in^44^. In recent years, many researchers have been using Firefly algorithm for optimization purposes^45^. The results of this algorithm for solving optimization problems have been more satisfactory than other algorithms including SA, GA, PSO, and HAS^46^. This meta-heuristic was inspired by firefly’s flashing and their communication^44^. There are about 2000 firefly species, and most of them produce rhythmic and short flashes^47^. Similar to other swarm intelligence algorithms whose details can capture a problem solution, in this algorithm each firefly is a solution to a problem. The light intensity also matches the objective function value. Notably, a firefly which has a brighter light is the solution and this firefly absorbs others.

#### Imperialist Competitive Algorithm (ICA)

**Figure 8:**
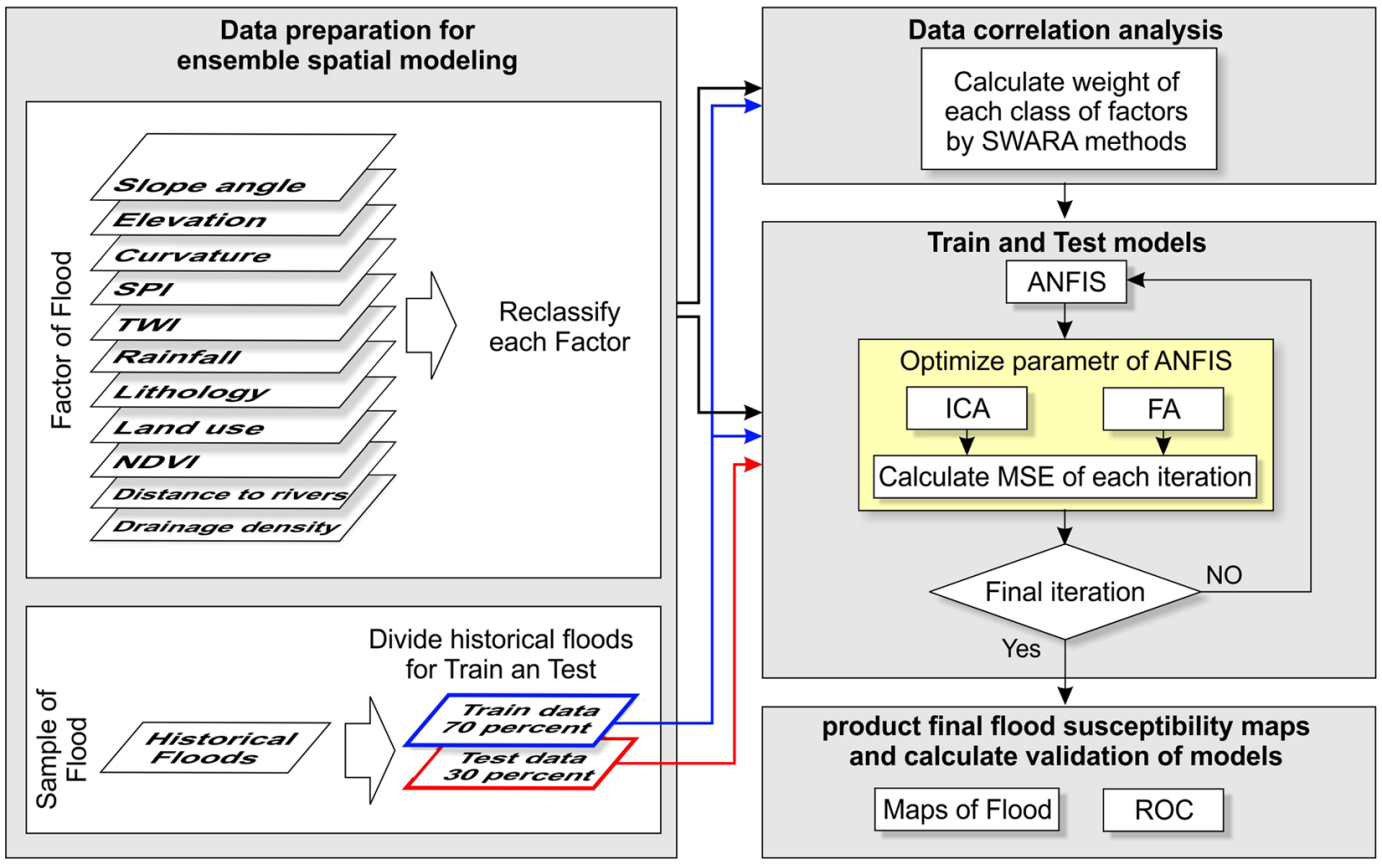
A general flowchart for optimization modelling in the study area.

One of the novel evolutionary algorithms developed according to socio-political relations is the colonial competition, introduced originally by Atashpaz-Gargari and Lucas^48^. Nowadays, ICA is known as a rich meta-heuristic algorithm and is used for optimization^49^. As with other evolutionary algorithms, ICA is made up of a series of components each indicating a solution to problems trying to find the best solution. A general flowchart of flood susceptibility mapping can be seen in Fig. 8.

### Evaluation and comparison methods

#### Statistical error-index

Evidently, when a new hybrid model is introduced, the performance of models should be evaluated and compared for both training and testing datasets. Basically, the results of modelling using a training dataset represent the degree of fit of models, while the results of a testing/validation dataset indicate the predictive power of modelling^30^.

In this study, root mean square error (RMSE) and mean square error (MSE) were applied as statistical error evaluation criteria along with ROC curve for evaluating and comparing the performance of the two flood hybrid models. RMSE and MSE can be calculated as^50^:

7 $${\rm{RMSE}}=\sqrt{\frac{\mathop{\sum }\limits_{i=1}^{n}{({{\rm{X}}}_{{\rm{est}}}-{{\rm{X}}}_{{\rm{obs}}})}^{2}}{{\rm{N}}}}$$

8 $$\text{MSE}=\frac{1}{{\rm{n}}}\mathop{\sum }\limits_{i=1}^{n}{({{\rm{X}}}_{{\rm{est}}}-{{\rm{X}}}_{{\rm{obs}}})}^{2}$$

where, *X*_*est*_ denotes the value estimated by a model, *X*_*obs*_ is the actual value (observed), and n shows the number of observations in the dataset.

#### Receiver operating characteristic curve

Receiver Operating Characteristic (ROC) is another standard technique to determine the general performance of models, which has been used in geoscience^51^. It is constructed by plotting the values of two statistical indexes, “sensitivity” and “100-specificity”, on the y-axis and x-axis, respectively^52^. Based on the definition, the number of positive cases (flood) which are correctly classified as positive (flood) class refers to sensitivity, while 100-specificity is considered as the number of negative (non-flood) cases correctly classified as negative (non-flood) class.

The area under the ROC curve (AUROC) quantitatively evaluates the models’ performance and their capability of predicting an event’s occurrence or non-occurrence^53^. The AUROC represents how well a flood model generally performs. It ranges between 0.5 (inaccuracy) and 1 (perfect model/high accuracy), where the higher the AUROC, the better the model performance is^54^. The AUROC can be formulated as:

9 $$AUROC=\frac{\sum TP+\sum TN}{P+N}\,$$

where, TP refers to the percentage of positive instances which are classified correctly. TN shows the percentage of negative instances which are classified correctly. Then, P denotes the total number of events (flood), and N is the total number of no-events (non-flood).

#### Non-parametric statistical tests

The performance of the two new hybrid models for flood modelling was assessed by parametric and non-parametric statistical tests^24^. If the data follows a normal distribution with an equal variance, parametric methods are applied^55^. Although non-parametric tests are free from any statistical assumptions, they are safer and their results are stronger than those of parametric tests, since the former do not assume normal distribution or homogeneity of variance^56^. Since the conditioning factors have been classified as categorical classes, non-parametric statistical tests should be used. For this reason, Freidman^57^ and Wilcoxon^58^ sign rank tests were used to assess the differences between treatments of hybrid models. The objective of these tests was to reject or accept the null hypothesis stating that the performances of flood hybrid models were not different at the significance level of α = 0.05 (or 5%).

The Friedman test was first used for the data to compare the significant differences between two or more models. Bui *et al*.^54^ reported that in the Friedman test, if p-value is less than 0.05, then the result is not applicable and we are not able to judge the significant differences between two or more models^54^. Therefore, the Wilcoxon signed-rank test was applied to specify the statistical significance of systematic pairwise differences between the two flood hybrid models using two criteria including p-value and the z-value. Eventually, the null hypothesis was rejected according to the p-value < 0.05 and z-value exceeding the critical values of z (−1.96 and +1.96). Accordingly, the performance of the susceptibility models differed significantly^54^.
