# Author Correction: Novel Hybrid Evolutionary Algorithms for Spatial Prediction of Floods

**DOI:** 10.1038/s41598-021-93957-4

**Published:** 2021-07-20

**Authors:** Dieu Tien Bui, Mahdi Panahi, Himan Shahabi, Vijay P. Singh, Ataollah Shirzadi, Kamran Chapi, Khabat Khosravi, Wei Chen, Somayeh Panahi, Shaojun Li, Baharin Bin Ahmad

**Affiliations:** 1 Geographic Information Science Research Group, Ton Duc Thang University, Ho Chi Minh City, Vietnam; 2 Faculty of Environment and Labour Safety, Ton Duc Thang University, Ho Chi Minh City, Vietnam; 3 Geohazard Department Manager, Samaneh Kansar Zamin (SKZ) Company, Tehran, Iran; 4 Department of Geomorphology, Faculty of Natural Resources, University of Kurdistan, Sanandaj, Iran; 5 Department of Biological and Agricultural Engineering & Zachry Department of Civil Engineering, Texas A & M University, College Station, TX 77843-2117 USA; 6 Department of Rangeland and Watershed Management, Faculty of Natural Resources, University of Kurdistan, Sanandaj, Iran; 7 Department of Watershed Management, Faculty of Natural Resources, Sari Agricultural Science and Natural Resources University, Sari, Iran; 8 College of Geology & Environment, Xi’an University of Science and Technology, Xi’an, 710054 Shaanxi China; 9 Young Researchers and Elites Club, North Tehran Branch, Islamic Azad University, Tehran, Iran; 10 State Key Laboratory of Geomechanics and Geotechnical Engineering, Institute of Rock and Soil Mechanics, Chinese Academy of Sciences, Wuhan, 430071 Hubei China; 11 Department of Geoinformation, Faculty of Geoinformation and Real Estate, Universiti Teknologi Malaysia (UTM), Johor Bahru, Malaysia

Correction to: *Scientific Reports* 10.1038/s41598-018-33755-7, published online 18 October 2018

This Article contained errors.

In the Introduction, in the sentence:

“Each year flooding affects around 200 million people and causes economic losses of about $95 billion around the world^3^.”

Reference 3 was to Chapi et al. 2017. It should direct the reader to Cealo et al. 2014. This has now been corrected, and the references were re-ordered as a result. Cealo et al. is now the cited Reference 3 in this sentence, and Chapi et al.—Reference 24, cited elsewhere in the article.

In the section “Description of the Study Area”

“The average annual rainfall is around 770 mm.”

now reads:

“The average annual rainfall of Mazandaran province is 770 mm, and for the Haraz watershed case study, it is 430 mm.”

There was a typographical error in the last row of Table 1, where class “535–471” now reads “535–741”. This table contains data published contemporaneously in another publication from the authors. This is now acknowledged in the table legend, which reads:

“Spatial relationship between flood and conditioning factors of SWARA model. Table republished from Reference 27.”

In the Materials and Methods section, subheading ‘Data collection and preparation’.

“Additionally, DEM and geological maps in the Haraz watershed were also used in data collection and preparation.”

now reads:

“Additionally, DEM (derived from the Advanced Spaceborne Thermal Emission and Reflection Radiometer (ASTER) Global DEM (https://gdex.cr.usgs.gov/gdex/) with a resolution of 30 × 30 m), and geological maps in the Haraz watershed were also used in data collection and preparation.”

Figure 1 displayed incorrect scale (the scale bar itself was correct). The incorrect scale is now removed.

Figure 2 contained images obtained from press sources and re-used without appropriate permissions. Since the original source cannot be established and these were used for demonstrative purposes only (to visualise the severity of the floods for the readers), the figure was now removed. Additionally, original Fig. 3 was now moved towards the end of the Article. Figures 3–9 were renumbered accordingly.

These changes do not affect the conclusions of the Article.

